# Safety and effectiveness results of an innovative injectable poly‐L‐lactic acid‐based collagen stimulator (Lanluma®)—Clinical outcomes at 9 months in a post‐market study

**DOI:** 10.1111/jocd.16527

**Published:** 2024-09-04

**Authors:** Moises Amselem, Dolly Fatsea, Riccardo Forte, Carlo Hasenöhrl, Ariel Haus, Ali Saalabian

**Affiliations:** ^1^ Private practice Madrid Spain; ^2^ Private practice Athens Greece; ^3^ Private practice Milan Italy; ^4^ Private practice Innsbruck Austria; ^5^ Private practice London United Kingdom; ^6^ Private practice Vienna Austria

**Keywords:** collagen stimulator, cosmetic techniques, poly‐L‐lactic acid (PLLA), safety, treatment outcomes

## Abstract

**Background:**

Injectable fillers for soft tissue augmentation stand out as one of the most favored procedures in the field of aesthetic medicine, especially in addressing the clinical signs of skin aging. Among soft tissue fillers, non‐permanent fillers have been safely used in numerous medical applications for several decades.

**Aims:**

The aim of this post‐market observational, open‐label, uncontrolled, multicentered, prospective study (PMS) was to evaluate the effects of an injectable poly‐L‐lactic acid‐based collagen stimulator (Lanluma**®**, the study product).

**Participants/Methods:**

This analysis is based on the clinical outcomes data (safety and effectiveness) collected from investigators and participants between the first injection (T0, September 2022) and 9 months thereafter (T3, June 2023) in the treatment of five body‐contouring areas.

**Results:**

Overall, 70 participants had 99 treatment sessions of the neck (31%), upper arm (20%), hand (17%), thigh (16%) and décolleté (15%). Lumps (neck, upper arm, hand) and nodules (neck, hand, thigh) were the most frequent adverse events (AEs) reported by investigators. All were treatment related. None were serious, severe or fatal. No AEs were reported following treatment of the décolleté. Both investigators and participants reported high levels of satisfaction during the nine‐month follow‐up period with the treatments in five body areas.

**Conclusions:**

These positive clinical outcomes can be attributed to a proper implementation of best practices and recommendations, and the rheological properties of the study product. This 9‐month follow‐up analysis should be reconsidered in light of the study's objectives for the final analysis at the 25‐month follow‐up.

## 1 INTRODUCTION

Injectable fillers for soft tissue augmentation stand out as one of the most favored procedures in the field of aesthetic medicine, especially in addressing the clinical signs of skin aging. The American Society of Plastic Surgeons estimated that the number of soft tissue filler injections in the US reached 3.4 million in 2020, showing a remarkable 422% increase from the year 2000.^1^ In 2020, the specific type of collagen stimulators represented 12% of all soft tissue fillers products. This rise in popularity can be attributed to consumer preference for minimally invasive procedures which offer the advantages of reduced pain and shorter recovery periods compared to conventional surgery. Additionally, the growing range of product types and indications, coupled with the influence of popular culture and social media promoting the concept of perpetual youthfulness, has contributed to the trend.^2^, ^3^ Benefiting from that growth, knowledge about the mechanisms of injectable fillers' aesthetic and biological effects has expanded^4^ simultaneously, with practices becoming safer.^5^, ^6^

Among soft tissue fillers, non‐permanent fillers are gradually metabolized by the body over a span of 6 to 24 months.^4^ Currently, the most common semi‐permanent fillers are hyaluronic acid (HA), calcium hydroxylapatite (CaHA) and poly‐L‐lactic acid (PLLA). While HA primarily “fills” the injected area, CaHA and PLLA induce a mild inflammatory response that stimulates the growth of fibroblasts and the synthesis of collagen. This gradual process leads to a sustained increase in dermal volume.^4^

PLLA has been safely used in numerous medical applications for several decades.^7^, ^8^ In the context of aesthetic medicine applications, injectable PLLA delivers results that manifest gradually and last for more than 2 years.^9^ Furthermore, the use of PLLA products is governed by the dissemination of best practices and expert recommendations.^10^, ^11^, ^12^, ^13^

Evidence of the safety and effectiveness of PLLA‐based collagen stimulators is primarily derived from observing real‐life practices.^7^, ^14^, ^15^, ^16^ Both adverse events (AEs) and the effectiveness of injectable PLLA have been documented.^8^, ^13^ Initially, PLLA‐based collagen stimulators were indicated for treating various facial areas^8^, ^9^, ^14^, ^15^ and clinical practice expanded to include other parts of the body, satisfying patient demand for rejuvenation of, among others, the neck and chest areas and further arm, hand and thigh areas.^7^, ^15^, ^16^ Safety and effectiveness data were therefore first documented from facial use and then gradually explored for other body areas. As a result, there is a substantial amount of literature for facial applications. While information is scarcer for the neck and chest areas, few studies have reported outcomes for treatments of the arm, hand and thigh areas. These studies have limited external validity since they were either case reports or retrospective studies.^7^

Amid the extensive array of soft tissue fillers available, conducting clinical outcome studies would enable practitioners to make informed product selection decisions, consequently benefiting their patients. This post‐market study (PMS) evaluated the effects of an injectable PLLA‐based collagen stimulator (Lanluma**®**, hereafter the study product). The objective was to gather additional data on clinical outcomes and assess the product's safety and effectiveness.

## 2 MATERIALS AND METHODS

This observational, open‐label, uncontrolled, multicentered, prospective study was designed to assess the safety and effectiveness of the study product in the treatment of 5 body‐contouring areas over a 25‐month period. The study began in June 2022 and is planned to end in November 2024. This analysis was based on the clinical outcomes data collected between the first injection (T0, September 2022) and 9 months thereafter (T3, June 2023).

### 2.1 Participants

The targeted study population was female and male participants aged 40–65 years. Participants were enrolled from six European clinics (Greece, UK, Italy, Spain and two in Austria), where the product is routinely used. Candidate participants were identified following an assessment and decision made by the investigator physician, based on a set of inclusion and exclusion criteria (Table 1). This process also involved obtaining a signed informed consent form from each participant. Patient Unique Numbers (PUNs) were assigned to anonymize enrolled participants.

**TABLE 1:** Inclusion and exclusion criteria.

Inclusion criteria
Eligible subjects for neck, hands and arms include females and males aged 40–65 years inclusively across a range of Fitzpatrick skin types.
Eligible subjects for décolleté and thighs include females aged 40–65 years inclusively across a range of Fitzpatrick skin types.
The subjects will participate in the study after assessment and decision of the physician.
Participants must fully understand the contents of the informed consent form and sign and date the informed consent form.
Participants must be willing and able to attend follow‐up visits for assessment of safety/effectiveness
Participants must agree not to undertake any further aesthetic treatment adjacent to or in the treatment area prior to their final study assessment visit.
Exclusion criteria
Participants with foreign body reaction sensitivity or known or suspected allergies to PLLA or any of the other ingredients should not undergo this treatment.
Subjects who:
have active sepsis or infection
have active (or a history of) autoimmune disease
are under 18 years of age
are pregnant or breastfeeding women or plan to become pregnant during the study period
are unwilling or unable to follow post‐treatment recommendations
have received any other aesthetic procedures in the treatment area at any time during the study period
have been deprived of their freedom by administrative or legal decision or are under guardianship
in the opinion of the investigator are unsuitable to take part in the study for scientific or medical reasons
are currently enrolled in other clinical trials
have known allergies to the product ingredients (i.e., Poly‐L‐lactic Acid (PLLA), sodium carboxymethyl cellulose (CMC), Mannitol)
have a cutaneous disorder, inflammation, infection, significant scarring, open wounds, lesions or tattoos in the body area
take thrombolytics or anticoagulants
have bleeding disorders.
have a history of severe allergy or anaphylactic shock
have porphyria
tend to form keloids, hypertrophic scars or any other healing disorders

Participants were briefed on various aspects of the study, ensuring their willingness to participate in photo documentation, and including timely completion and sharing of the questionnaire. They were also advised to wait for several sessions (2–3 months apart) to observe improvements in skin quality and texture from the treatment.

### 2.2 Materials

The study product (Lanluma®), a class III medical device bearing CE marking (CE 1023), was introduced in February 2021. This injectable filler is made of PLLA, a synthetic, biodegradable, biocompatible and immunologically inert polymer belonging to the alpha‐hydroxy acid family. Its purpose is to enhance the volume of sunken skin areas, notably to correct skin depressions. The study product is recommended for the treatment of buttocks, abdomen, upper arms, thighs, hands, neck, décolleté, face and cellulite. The study product is supplied in the form of a sterile freeze‐dried preparation for injection which, after reconstitution with sterile water, yields a 210 mg/15 mL (14 mg/mL) solution for injection. After slowly adding the recommended volume of sterile water, the vial is shaken for 10 min and let stand for at least 1 h to ensure complete hydration. The reconstituted product is usable within 72 h of reconstitution and any material remaining after use or after 72 h following reconstitution is discarded. Immediately prior to use, the reconstituted product is doubled diluted to 7 mg/mL and should be strongly agitated for 1 min, until a uniform translucent suspension is obtained (Table 2).

**TABLE 2:** Recommended accessories and safe volumes for injection of the study product.

Area	Volume of study product	Dilution	Device
Neck	2 mL per side	Double dilution [30 mL]	22G–23G cannula
Décolleté	10 mL up to 15 mL per side	Double dilution [30 mL]	23G cannula
Hands	Up to 1 mL per hand	Double dilution [30 mL]	21G–22G cannula
Upper arm	Up to 1 vial^a^ per arm	Double dilution [30 mL]	21G–23G cannula
Thighs	1–2 vials^a^ per leg	Double dilution [30 mL]	21G cannula

^a^ 1 vial of study product contains 210 mg PLLA. Instructions for use available from https://eifu.sinclairpharma.com/LAN and based on internal training manual.

Both investigators and participants received specific instructions for post‐injection massage in line with the product information for use. Investigators were required to massage each treated area for 1–2 min using a rotational technique before proceeding to the next area. Participants were informed of their responsibility to massage the treated areas for 5 min, five times a day, for at least 2 weeks.

### 2.3 Treatment indications

Five treatment indications were assessed within this PMS: improved skin laxity in the mid neck, wrinkle reduction at the décolleté area, improved skin laxity and anti‐aging in hands, improved skin laxity and uniformity of appearance in thighs, and improved skin laxity and uniformity of appearance (smoother skin) in upper arms.

The treatment areas were chosen by the participant in consultation with the investigator. Reconstitution and treatment were required to be performed according to the predefined injection protocols per indication and in accordance with the product's instructions for use (Table 2). Three treatment sessions of the individual body areas were recommended, at the initial visit (T0), after 3 months (T1) and after 6 months (T2). The third treatment was optional based on the results achieved.

### 2.4 Data collection and objective

The study data were collected by the investigators via questionnaires and standardized photographs at predefined timepoints and participant visits (Table 3). Participants will be followed up over a total period of 25 months.

**TABLE 3:** Schedule of assessment.

Visit	Date	Injection	Safety	Effectiveness	Satisfaction	Picture
Participant recruitment	2022 August–September					
1st visit (T0)_Initial visit	2022 September	x	x		x	x
2nd visit (T1)	2022 December	x	x			x
3rd visit (T2)	2023 March	x	x	x	x	x
4th visit (T3)	2023 June		x	x		x
5th visit (T4)	2023 December		x	x	x	x
6th visit (T5)	2024 June		x	x		x
7th visit (T6)–FINAL visit	2024 October		x	x	x	x

The main objective of the study is to assess the safety and effectiveness of the study treatment when used in standard clinical practice following standardized injection protocols.

#### 2.4.1 Safety assessments

Safety data were recorded by the investigator during study visits using a standardized questionnaire at specific intervals: the day of initial treatment (T0), 3 months (T1), 6 months (T2), 9 months (T3), 12 months (T4), 18 months (T5) and 24 months (T6) after treatment. For each individual AE, the following information was documented: Start date, End date, Seriousness, Relationship with treatment, Severity, Concomitant medication given, and Outcome of event.

Safety data were also collected through another standardized questionnaire completed by the participants themselves during study visits. For each reported complication, participants were asked to indicate the appropriate outcome related to the following: None, Start date, End date, Ongoing, Mild, Moderate, Severe.

Both investigators' and participants' questionnaires included assessment of the following events: Itching, Pain/discomfort, Lumps, Nodules, Redness, Localized infection, Swelling, as well as any other events reported in two open fields when applicable. Lump were defined as non‐inflamed papule created by product and nodule were defined as inflamed bump which can be red or painful.

The primary safety endpoint was the collection of all AEs documented by the investigator from baseline to the end of the study (25 months). The secondary safety endpoints were the evaluation of clinical safety as assessed by a standardized questionnaires completed by the investigator and by the participant at the day of injection and 3, 6, 9, 12, 18 and 24 months thereafter.

#### 2.4.2 Effectiveness assessments

Effectiveness data were recorded by the investigator based on predefined rating scales for individual treated indications—Neck (validated five‐grade Neck Laxity Scale), Décolleté (Validated Assessment Scales for Décolleté Wrinkling and Pigmentation), Hands (a validated five‐point scale to grade appearance of the dorsum of the hand), Arms (five‐grade Arm Visual Analog Scale [Arm VAS]) and Thighs (five‐grade Photonumeric grading scale). The visual effect of the study product was documented through photographs taken at the predefined timepoints T0 to T6 and then compared to baseline photographs. The clinical evaluation of the treatment was assessed with the Global Aesthetic Improvement Scale (GAIS).

The primary efficacy endpoint was the percentage of participants showing improvements in their respective treated indications, as assessed by predefined rating scales.

## 3 RESULTS

### 3.1 Study population and treatments

At the time T3, 9 months after the first injection, 70 participants were followed up. The average age at inclusion was 55 years old (min: 40 years old—max: 66 years old) and 89% were females.

Overall, 99 treatment sessions were administered, with some participants having 2 body areas injected (on average 1.4 indications per participant). The breakdown of indications was primarily the neck (31%, *n* = 31) and upper arm (20%, *n* = 20), followed by the hand (17%, *n* = 17), thigh (16%, *n* = 16) and décolleté (15%, *n* = 15).

### 3.2 Safety assessment at 9 months

**FIGURE 1:**
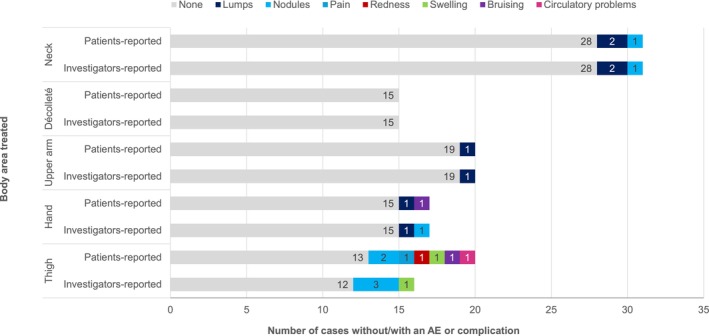
Safety assessment 9 months after the first injection (T3), participant‐reported complications and investigator‐reported adverse events. *N* = 70 participants receiving *n* = 99 treatment sessions, neck (*n* = 31), décolleté (*n* = 15), upper arm (*n* = 20), hand (*n* = 17), thigh (*n* = 16). Reading: Over the course of 31 treatment sessions of the neck area, no adverse events were reported in 28 treatment session, lumps occurred in 2 treatment sessions and nodule occurred in 1 treatment session.

Over 9 months after the first injection (T3), 10 AEs were reported by the investigators (10% of treatment sessions) and 13 complications (13% of treatment sessions) were reported by the participants. Lumps and nodules were the most frequent AEs or complications reported by investigators and participants. Other AEs were identified as treatment‐related and classified as mild or moderate and non‐serious in one single patient who reported “other” defined as follows: pain (moderate), redness (moderate), swelling (mild), bruises (moderate) and circulatory problems (moderate, with dizziness and cold sweats). The treatment was continued, the complications were resolved the next day, except the nodules which was ongoing at reporting timepoint (Figure 1).

AEs and complications were observed following injections in the neck, upper arm, hand and thigh, with none reported following treatment of the décolleté (Figure 1). None of the treatments initiated was discontinued due to an AE or complication.

Investigators reported AEs after injections in the neck: two cases of mild non‐serious lumps lasting for 7 days recovered without additional intervention or medication; one instance of non‐serious moderate nodules had been ongoing for 5 months at T3. Participants' reports of complications were consistent with investigators' observations.

Following treatment of the thigh area, investigators reported three cases of mild non‐serious nodules. At the time of observation, two had resolved after 14 days and 59 days and one case was unresolved after 36 days. Participants reported two cases of nodules of moderate intensity, persisting for 2–6 weeks at the time of observation. Under the category of “others”, a single participant reported moderate pain, moderate redness, mild swelling, moderate bruising and moderate circulatory problems. These complications resolved after a few days and after a few weeks for the bruising.

Investigators reported one case of a nodule and one case of a lump following treatment in the hands, while participants themselves reported experiencing a lump and bruising (one case each) in the same area. Investigators and participants reported a single case of a nodule after treatment of the upper arm and no AE or complication was reported after treatment of the décolleté area (Figure 1).

### 3.3 Participant assessment of effectiveness

**FIGURE 2:**
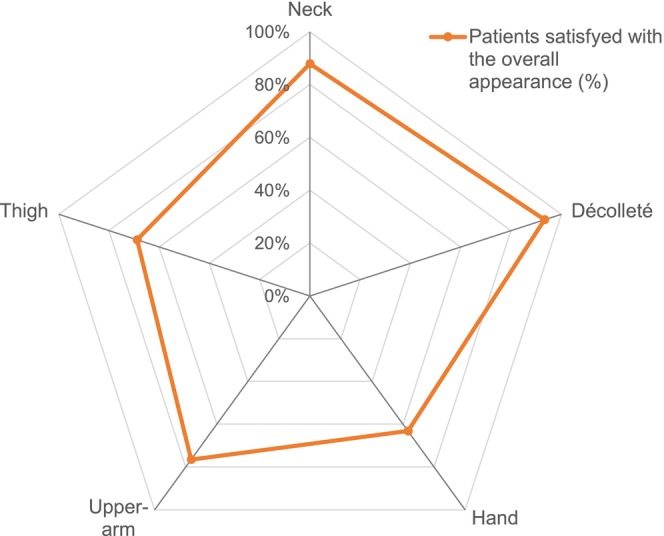
Participants' satisfaction evaluated 9 months after the first injection (T3). Participants' satisfaction was evaluated based on their level of agreement with the statement “I am satisfied with the overall appearance of my [body area]” using the responses: Totally agree, Agree, Somewhat agree, Somewhat disagree, Disagree and Totally disagree. The subtotal of Totally agree, Agree and Somewhat agree responses is represented.

A significant number of participants reported satisfaction with the overall appearance of their treated body areas at T3. Proportions of participants who agreed, from “totally” to “somewhat,” with the statement “I am satisfied with the overall appearance of my […] area” were 93.3% for the décolleté area, 87.9% after treatment of the neck and 76.5% for the upper arm. Slightly fewer participants were satisfied after treatment of the thigh area (68.8%) and the hand area (63.2%) (Figure 2).

All aspects assessed by participants were highly satisfactory regarding treatment of the décolleté and neck. For instance, the majority of participants were satisfied with their skin texture after treatment, agreeing that there was an improvement with reduced wrinkles and a more youthful look (Table 4). For the neck area, 93.6% reported an improved texture and 93.5% were satisfied with the smoothness of their skin. Moreover, 86.7% of participants reported increased self‐confidence after treatment of their décolleté area, while 83.9% expressed the same for their neck area (Table 4).

**TABLE 4:** Satisfaction reported by participants at T3.

[Body area]	Answer	Satisfied with the overall appearance (%)	Satisfied with smoothness (%)	Satisfied with skin texture thickness/firmness (%)	Feeling more confident (%)	Improved look of the skin's texture (%)	More youthful look (%)	Reduced wrinkles (appearance) (%)
Neck	Totally agree	41.9	41.9	38.7	41.9	35.5	32.3	32.3
	Agree	32.3	29.0	29.0	32.3	35.5	35.5	32.3
	Somewhat agree	12.9	22.6	22.6	9.7	22.6	16.1	22.6
	ST AGREE	87.1	93.5	90.3	83.9	93.6	83.9	87.2
	Somewhat disagree	12.9	6.5	9.7	12.9	3.2	12.9	6.5
	Disagree	0.0	0.0	0.0	3.2	3.2	3.2	6.5
	Totally disagree	0.0	0.0	0.0	0.0	0.0	0.0	0.0
Upper arm	Totally agree	25.0	30.0	30.0	30.0	35.0	20.0	‐
	Agree	15.0	10.0	10.0	15.0	5.0	25.0	‐
	Somewhat agree	25.0	30.0	25.0	15.0	25.0	20.0	‐
	ST AGREE	65.0	70.0	65.0	60.0	65.0	65.0	NA
	Somewhat disagree	20.0	15.0	20.0	25.0	20.0	20.0	‐
	Disagree	15.0	15.0	15.0	15.0	15.0	15.0	‐
	Totally disagree	0.0	0.0	0.0	0.0	0.0	0.0	‐
Hand	Totally agree	35.3	‐	35.3	35.3	35.3	41.2	35.3
	Agree	29.4	‐	29.4	17.6	23.5	17.6	29.4
	Somewhat agree	11.8	‐	23.5	17.6	35.3	17.6	11.8
	ST AGREE	76.5	NA	88.2	70.5	94.1	76.4	76.5
	Somewhat disagree	17.6	‐	5.9	23.5	0.0	17.6	17.6
	Disagree	5.9	‐	5.9	5.9	5.9	5.9	5.9
	Totally disagree	0.0	‐	0.0	0.0	0.0	0.0	0.0
Décolleté	Totally agree	53.3	46.7	73.3	53.3	53.3	60.0	80.0
	Agree	40.0	33.3	13.3	26.7	40.0	33.3	13.3
	Somewhat agree	0.0	6.7	6.7	6.7	0.0	0.0	0.0
	ST AGREE	93.3	86.7	93.3	86.7	93.3	93.3	93.3
	Somewhat disagree	0.0	6.7	0.0	6.7	0.0	0.0	0.0
	Disagree	6.7	6.7	6.7	6.7	6.7	6.7	6.7
	Totally disagree	0.0	0.0	0.0	0.0	0.0	0.0	0.0
Thigh	Totally agree	12.5	‐	18.8	12.5	12.5	12.5	12.5
	Agree	31.3	‐	37.5	37.5	31.3	18.8	31.3
	Somewhat agree	25.0	‐	18.8	12.5	31.3	12.5	18.8
	ST AGREE	68.8	NA	75.1	62.5	75.1	43.8	62.6
	Somewhat disagree	25.0	‐	12.5	18.8	6.3	37.5	18.8
	Disagree	0.0	‐	0.0	0.0	6.3	6.3	0.0
	Totally disagree	6.3	‐	12.5	18.8	12.5	12.5	18.8

Abbreviations: NA, not applicable; ST, subtotal.

Shades are meant to highlight the sub‐total (ST) "AGREE".

Satisfaction levels following hand treatment exhibited greater diversity across the evaluated aspects. The satisfaction rate for skin texture was notably high (88.2%), with 94.1% indicating an improved look. The perception of a youthful appearance received a satisfactory rating of 76.4%, while 76.5% reported a reduction in wrinkles and 70.5% expressed increased self‐confidence (Table 4).

Satisfaction levels after upper arm and thigh treatment, while still positive, were slightly lower in terms of improved skin texture and appearance (65.0% and 75.1%, respectively), satisfaction with skin texture (65.0% and 75.1%), achieving a more youthful appearance (65.0% and 43.8%, respectively) and increasing self‐confidence (60.0% and 62.5%) (Table 4).

### 3.4 Investigator assessment of effectiveness

**FIGURE 3:**
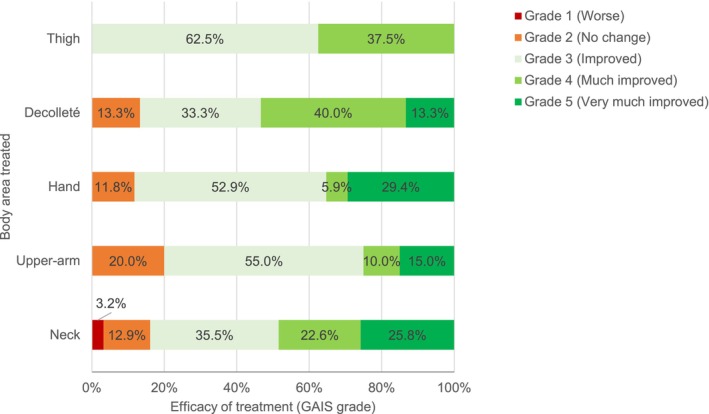
Effectiveness assessment based on the Global Aesthetic Improvement Scale (GAIS), as assessed by investigators between T0 and 9 months after the first injection (T3). GAIS grades: Grade 1 (Worse—The appearance is worse than the original condition), Grade 2 (No change—The appearance is essentially the same as the original condition), Grade 3 (Improved—Obvious improvement in appearance from the initial condition), Grade 4 (Much improved—Marked improvement in appearance from the initial condition), Grade 5 (Very much improved—Excellent corrective result). *N* = 99 treatments.

The effectiveness assessed by investigators using GAIS grades indicates that most treatments improved the appearance of the treated area (Grade 3 or more). It is noteworthy that the percentages of investigators attributing the Grade 5 rating (reflecting excellent corrective results) were 25.8% for treatment of the neck area and 29.4% for treatment of the hand area (Figure 3).

The evaluations made by the investigators closely aligned with those provided by the participants. Treatment of the neck received remarkable ratings, with 87.1% of investigators agreeing on smoother skin, 83.8% reporting a more youthful appearance and 87.1% noting an enhanced skin texture.

Regarding the décolleté area, 93.4% of investigators agreed on increased smoothness and 93.3% reported an improvement in skin texture and a more youthful appearance.

Substantial percentages of investigators also reported satisfactory smoothness (94.2%), improved skin texture (82.3%) and a more youthful appearance (76.5%) after treatment of the hand area.

Lastly, after thigh and upper arm treatments, the investigator‐reported improvements were as follows: 87.5% and 85.0% for smoothness, 75.1% and 75.0% for improved skin texture, and 81.3% and 70.0% for a more youthful look (Table 5).

**TABLE 5:** Satisfaction reported by investigators based on standardized T3 questionnaires.

[Body area]	Answer	Improved texture of the skin (%)	Skin looks smoother (tighter and firmer) (%)	More youthful look (%)
Neck	Totally agree	38.7	51.6	54.8
	Agree	32.3	19.4	12.9
	Somewhat agree	16.1	16.1	16.1
	ST AGREE	87.1	87.1	83.8
	Somewhat disagree	6.5	6.5	9.7
	Disagree	6.5	6.5	6.5
	Totally disagree	0.0	0.0	0.0
Upper arm	Totally agree	25.0	25.0	30.0
	Agree	20.0	15.0	15.0
	Somewhat agree	30.0	45.0	25.0
	ST AGREE	75.0	85.0	70.0
	Somewhat disagree	15.0	5.0	10.0
	Disagree	10.0	10.0	20.0
	Totally disagree	0.0	0.0	0.0
Hand	Totally agree	41.2	47.1	47.1
	Agree	23.5	11.8	11.8
	Somewhat agree	17.6	35.3	17.6
	ST AGREE	82.3	94.2	76.5
	Somewhat disagree	17.6	5.9	23.5
	Disagree	0.0	0.0	0.0
	Totally disagree	0.0	0.0	0.0
Décolleté	Totally agree	46.7	66.7	60.0
	Agree	33.3	20.0	20.0
	Somewhat agree	13.3	6.7	13.3
	ST AGREE	93.3	93.4	93.3
	Somewhat disagree	6.7	6.7	6.7
	Disagree	0.0	0.0	0.0
	Totally disagree	0.0	0.0	0.0
Thigh	Totally agree	25.0	12.5	18.8
	Agree	31.3	50.0	37.5
	Somewhat agree	18.8	25.0	25.0
	ST AGREE	75.1	87.5	81.3
	Somewhat disagree	25.0	12.5	18.8
	Disagree	0.0	0.0	0.0
	Totally disagree	0.0	0.0	0.0

Abbreviations: NA, not applicable; ST, subtotal.

Shades are meant to highlight the sub‐total (ST) "AGREE".

## 4 DISCUSSION

This analysis provides safety and effectiveness data for an innovative injectable PLLA product (Lanluma**®**) used to treat five body areas—neck, décolleté, upper arm, hand and thigh—indications that have received less attention compared to the face.

As per the American Society of Plastic Surgeons 2020 statistics^1^ on PLLA injections specifically, 57% fall in the 40–54 age range and 25% in the 55–69 age range. Additionally, women account for 83% of all PLLA injections performed. The study population therefore reflects the demographic composition of the broader population receiving PLLA injections.

The overview of investigator‐reported AEs, consistent with participant‐reported complications, indicates that all indications were safe. Most AEs and complications were non‐serious nodules and lumps of mild intensity, which resolved spontaneously. They were reported following treatment of the neck, hands, upper arm and thigh areas, while none occurred after injection of the décolleté. It is recognized that nodules are commonly reported AEs associated with PLLA injections,^7^, ^17^, ^18^ particularly in body areas with less subcutaneous fat compared to the face, such as the neck or hands.^15^, ^17^ However, their occurrence is mainly related to the injection technique and uneven distribution of the product in the tissue.^13^ Experience with PLLA‐based products has significantly reduced the incidence of nodules to less than 10% of participants, with most cases resolving spontaneously.^18^, ^19^

In this intermediate analysis over 99 treatment sessions, 9 months after the first injection, investigators reported 10 AEs, including 5 cases of nodules. The low incidence of AEs and complications suggests that the observed practices were in line with recommendations and demonstrated a good understanding of the study product. Notably, the administered volumes were likely sufficient to avoid the formation of nodules.^7^, ^17^ Nonetheless, it is advisable to remain attentive to injection techniques and post‐injection massages^13^, ^20^ to further mitigate the emergence of nodules.

The effectiveness assessment at nine‐month follow‐up shows high levels of satisfaction among participants and investigators. A maximum of three treatment sessions were administered per indication, the third one being optional based on the result achieved. All indications received high degrees of satisfaction from both general and individual appreciations of the smoothness, texture or appearance of the skin.

The literature consistently reflects elevated participant satisfaction with PLLA injections.^3^, ^7^ In fact, prevailing practices stem from widely shared recommendations that underscore thorough participant selection, the administration of treatment by skilled and experienced physicians, and adherence to product preparation and administration instructions. It is also recommended to avoid over‐corrections and to emphasize post‐injection massages of the treated area.^7^

In the case of the study product, precise injection protocols are provided to physicians and skills are continually supported through training sessions. The reconstitution of the product is also critical, linked to the homogeneity of the injected solution. The study product underwent a comparative study evidencing that the suspension after reconstitution remains visually homogeneous after 2 min and even after 30 min, while the comparator showed phase separation.^21^

Treatment of the thighs was notable for a lower level of satisfaction. This indication had the highest proportion of Grade 3 scores on the GAIS scale, with fewer Grades 4 (much improved) and 5 (very much improved). Similarly, treatment of the upper arm area was primarily evaluated with Grades 2 and 3, and less with Grades 4 and 5.

Interestingly, the thigh and upper arm areas, among those studied, are the ones most likely to be marked by cellulite or fat accumulation. Treatment objectives for these areas generally involve addressing skin laxity, cellulite and fat accumulation. Additionally, areas marked by fat accumulation can be effectively treated through combinations or sequences of treatments that may enhance skin quality.^7^, ^16^ Importantly, participants receiving any other aesthetic treatment for the observed treated areas were excluded from the study. Therefore, it can be hypothesized that a single PLLA treatment may not achieve the highest level of satisfaction in individuals most affected by fat accumulation in the thigh or upper arm areas.

### 4.1 Limitations

This study has certain limitations. As an observational study, it lacks both a control group and participants randomization, and it is based on a limited number of participants. Additionally, a quantitative comparison with literature data is challenging due to significant variations among physicians in reconstituting the injected suspension, total volume injected and injection techniques. Lastly, this 9‐month follow‐up analysis should be reconsidered in light of the study's objectives for the final analysis at the 25‐month follow‐up.

## 5 CONCLUSION

The study product, an injectable PLLA (Lanluma®), exhibited minimal rates of adverse events, and both investigators and participants reported high levels of satisfaction during the nine‐month follow‐up period for the treatment in five body areas—neck, décolleté, upper arm, hand and thigh. AEs were observed in areas that have thinner fat layers; therefore, the treatment is less recommended in body areas with no adipose tissue. Improving the results obtained, especially in the arms and thighs, is possible by adapting the concentration of PLLA injected and the number of sessions to each participant. These positive clinical outcomes can be attributed to the combination of a proper implementation of best practices and recommendations and the rheological properties of the study product.

## AUTHOR CONTRIBUTIONS

All authors contributed to the design of the study (including the questionnaire), the implementation of the questionnaire and the collection of data. All authors discussed the results and contributed to the final manuscript.

## CONFLICT OF INTEREST STATEMENT

DF, RF, CH, AH, AM, AS received fees from Sinclair Pharma and contracted with the SKINBOX. AS is KOL for a product of APYX Medical.

## ETHICS STATEMENT

Participants signed an informed consent form. Data were processed, evaluated, and stored in anonymous form (individual PUC were assigned for each patient at T0) in accordance with applicable data protection regulations.

## Supporting information


**Data S1:** Questionnaires Investigator.

**Data S2:** Questionnaires Patient.

## Data Availability

The data that support the findings of this study are available on request from the corresponding author. The data are not publicly available due to privacy or ethical restrictions.
