# Supplementary material for: Safety and effectiveness results of an innovative injectable poly‐L‐lactic acid‐based collagen stimulator (Lanluma®)—Clinical outcomes at 9 months in a post‐market study
Source: J Cosmet Dermatol. 2024 Sep 4;23(12):3893–902. doi: 10.1111/jocd.16527 (PMC11626332; doi:10.1111/jocd.16527)
Supplement: Supplementary file 1 — Data S1: Questionnaires Investigator. [file JOCD-23--s002.pdf]

investigator	neck

STUDY ENDPOINT

CLINICAL EVALUATION

EFFICACY

please rate clinically according IBSA neck laxity scale

5-grade IBSA neck laxity scale

Rating (Neck)	Description
Grade 1	Normal trophism of the tissues of the neck
Grade 2	Mild laxity of the tissues of the neck
Grade 3	Moderate laxity of the tissues of the neck
Grade 4	Moderate to severe laxity of the tissues of the neck
Grade 5	Severe laxity of the tissues of the neck

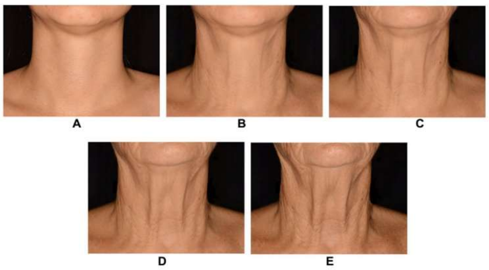

Figure 3 The 5-grade IBSA Neck Laxity Scale. (**A**) Grade 1: normal trophism of the tissues of the neck. (**B**) Grade 2: mild laxity of the tissues of the neck. (**C**) Grade 3: moderate laxity of the tissues of the neck. (**D**) Grade 4: moderate to severe laxity of the tissues of the neck. (**E**) Grade 5: severe laxity of the tissues of the neck.

please rate grade of severity according GAIS

Global Aesthetic Improvement Scale (GAIS)

Grade of Severity	definition
Grade 1	Worse The appearance is worse than the original condition
Grade 2	No change The appearance is essentially the same as the original
Grade 3	Improved Obvious improvement in appearance from the initial condition but a touch-up or re-treatment is indicated
Grade 4	Much improved Marked improvement in appearance from the initial condition, but not completely optimal for this subject.
grade 5	Very much improved Excellent corrective result

please rate skin quality

skin quality

	Totally agree	Agree	Somewhat agree	Somewhat disagree	Disagree	Totally disagree
The texture of the skin on the neck looks improved						
The skin on the neck looks smoother						
The neck looks more youthful						

SAFETY

please document adverse events

Adverse event	Start date	End date	Seriousness1	Relationship with treatment2	Severity3	Concomitant medication given	Outcome of event4
Itching							
Pain/discomfort							
Lumps							
Nodules							
Redness							
Localized infection							
Swelling							
another event 1 (please specify): ____________							
another event 2 (please specify): ____________							

[1] **Seriousness**	serious
	non-serious

**Related w**	unrelated
	related

[3] **Severity**	mild
	moderate
	severe

**concomitant medication**	yes
	no

[4] **Outcome of event:**	Recovered/resolved
	recovered/resolved with sequelae
	not recovered/not resolved
	resolving/recovering
	fatal
	unknown
