# Supplementary material for: Safety and effectiveness results of an innovative injectable poly‐L‐lactic acid‐based collagen stimulator (Lanluma®)—Clinical outcomes at 9 months in a post‐market study
Source: J Cosmet Dermatol. 2024 Sep 4;23(12):3893–902. doi: 10.1111/jocd.16527 (PMC11626332; doi:10.1111/jocd.16527)
Supplement: Supplementary file 2 — Data S2: Questionnaires Patient. [file JOCD-23--s001.pdf]

patient | neck

satisfaction

	Totally agree	Agree	Somewhat agree	Somewhat disagree	Disagree	Totally disagree
I am satisfied with the overall appearance of my neck area	◻	◻	◻	◻	◻	◻
I am satisfied with the improved appearance of smoothness of my neck	◻	◻	◻	◻	◻	◻
I am satisfied with the tone (thickness) of my neck area	◻	◻	◻	◻	◻	◻
I feel more confident after the treatment of my neck						
The texture of the skin on the neck looks improved						
The neck looks more youthful						
The wrinkles on my neck have been reduced						

safety

Please describe if any complication occured

Safety event	None	Start date	End date	Ongoing	Mild1	Moderate2	Severe3
Itching							
Pain/discomfort							
Lumps							
Nodules							
Redness							
Localized infection							
Swelling							
Another event 1 (please specify): ____________							
Another event 2 (please specify): ____________							
…………*							

*Option to add as many adverse events as required

[1]Mild: discomfort noted, but no disruption to normal daily activities.

[2]Moderate: discomfort sufficient to reduce or affect normal daily activities

[3]Severe: inability to work or carry out normal daily activities
